# Potential for increased prevalence of neuropathic pain after the COVID-19 pandemic

**DOI:** 10.1097/PR9.0000000000000884

**Published:** 2021-01-27

**Authors:** Nadine Attal, Valéria Martinez, Didier Bouhassira

**Affiliations:** a INSERM U 987, CETD, Hôpital Ambroise Paré, APHP, Boulogne-Billancourt, France; b Université Versailles Saint Quentin, Versailles, France; c Department of Anesthesiology and Pain Unit, Hôpital Raymond Poincaré, APHP, Garches, France

**Keywords:** COVID-19, SARS-CoV-2, Neurological complications, Neuropathic pain, Narrative review

## Abstract

Although coronavirus disease 2019 (COVID-19) most commonly manifests with acute respiratory symptoms, one very common symptom of COVID-19 is pain. As COVID-19 often causes peripheral or central neurological complications, it is anticipated that a number of the chronic pain complications of COVID-19 will be neuropathic. This review first examines the most common viral infections responsible for neurological complications including neuropathic pain. These encompass herpes zoster, HIV, poliovirus, enteroviruses, and several tropical viruses. Neurological complications of COVID-19 including in particular Guillain–Barré syndrome, myelitis, and stroke are reviewed with regards to their potential risk of chronic neuropathic pain. Prospective longitudinal cohorts of patients should be implemented to evaluate the exact risk of neuropathic pain after COVID-19.

## Abstract 2

This review focuses on the neurological complications of COVID-19 including Guillain–Barré syndrome, myelitis, and stroke with regards to their potential risk of chronic neuropathic pain.

## 1. Introduction

As of October 30, 2020, the coronavirus 19 disease (referred to as COVID-19) has infected more than 40 million people worldwide and caused 1.1 million deaths (World Health Organization). Although COVID-19 most commonly manifests with acute respiratory symptoms, one very common symptom of the disease is pain.^11^ Pain most commonly includes headache, joint pain, and muscle pain particularly at the acute phase,^15,66^ as is the case for other viral infections such as seasonal influenza or influenza A (H1N1).^33^ It has also been reported that patients with chronic pain infected with the severe acute respiratory syndrome coronavirus 2 (SARS-CoV-2) sometimes experience exacerbation of their symptoms, which may be due to multiple factors including social threats, discontinuation of therapy, or reduced access to treatments and concerns about health outcomes.^11,29^ The psychosocial impact of COVID-19 and of the lockdown in patients with chronic pain and the consequences in terms of therapeutic management have been outlined.^13,15–17,27,29,31,59^ By contrast, much less is known about the risk of newly developed long-term symptoms after COVID-19, sometimes referred to as long-covid, long haulers, or lingering manifestations^35,43,54^ which often include chronic pain.^11^ Chronic pain has been reported to emerge in relation to psychological stressors, the viral infection itself, or the consequences of admission to intensive care unit (ICU) and may include either regional or widespread pain.^11^ However, a special characteristic of COVID-19 is that it often causes peripheral or central neurological complications, either through direct invasion of the nervous system or through postviral immune reactions.^24,26,42,67^ Thus, beyond psychological stressors, we anticipate that some patients with chronic neuropathic pain exposed to the SARS-CoV-2 will develop more severe neurological complications, exacerbation of their neuropathic pain, or deterioration of their neurological condition. Other patients may present COVID-19–induced neuropathic pain because of neurological complications.

To date, the risk of increased or de novo neuropathic pain after COVID-19 and the potential specificities of COVID-19–related pain with regards to other viral infections has not been addressed. In this narrative review, we will first examine the risk of neurological lesions after viral infections other than the SARS-CoV-2 and the potential for neuropathic pain after COVID-19.

## 2. Viral infections and neuropathic pain

### 2.1. The nature of neurological complications

Viral infections may have a direct impact on the peripheral nervous system or central nervous system (CNS) or induce postviral immune syndrome. The most common peripheral lesions responsible for neuropathic pain include acute or chronic polyneuropathy, acute polyradiculoneuritis (Guillain–Barré syndrome), chronic inflammatory demyelinating polyneuropathy, or ganglionopathy. Guillain–Barré syndrome and chronic inflammatory demyelinating polyneuropathy in particular have been associated with a large number of viral agents including coronaviruses, Epstein–Barr virus, HIV, hepatitis virus, cytomegalovirus, influenza A virus, and Zika.^53^ Central nervous system lesions responsible for neuropathic pain after viral infections include transverse myelitis, encephalomyelitis, and stroke.

### 2.2. Viral infections most commonly responsible for neurological complications

#### 2.2.1. Herpes zoster

The most largely described neuropathic pain after viral infection is postherpetic neuralgia (PHN) which develops after herpes zoster caused by varicella zoster virus (VZV), a highly neurotropic virus (Table 1). Primary infection usually results in varicella (chickenpox), then VZV becomes latent in cranial nerve ganglia, dorsal root ganglia, and autonomic ganglia. Concomitant to age-related decline in cell-mediated immunity, the virus may be reactivated within a single or less commonly several ganglions to induce herpes zoster, characterized by rash and dermatomal-distribution.^22^ Postherpetic neuralgia occurs within 3 months after herpes zoster and represents the most common and burdensome complication of herpes virus, with a prevalence estimated at 6% to 10% in the year after herpes zoster.^28^ The main risk factors for PHN after herpes zoster are the severity of acute pain, older age, greater severity of infection, prodromal pain, and ophthalmic involvement.^7,20,28,51^ Pain is typically neuropathic and most commonly described as burning and paroxysmal, nearly constantly associated with severe allodynia to brush.^7^ It can be devastating in terms of quality-of-life impact particularly in the elderly.^7^ Treatment is difficult and is generally similar to other neuropathic pain syndromes,^19^ but 2 vaccines have been found effective in preventing herpes zoster and PHN.^21^

**Table 1:** Mechanisms of neuropathic pain in patients with viral infections.

Virus most commonly responsible for neurological lesions	Neurological lesion
Herpes zoster	Lesion of sensory ganglia (responsible for postherpetic neuralgia) Myelitis
HIV	Painful sensory polyneuropathy Myelitis
Enteroviruses	Myelitis
Poliovirus	Postpolio syndrome
HTLV1	Myelitis
Zika	Guillain–Barré syndrome
Chikungunya	Myelitis
Other viruses*	Guillain–Barré syndrome
COVID-19	Guillain–Barré syndrome Myelitis Stroke Encephalitis

* eg, Epstein–Barr, cytomegalovirus, influenza A, coronaviruses, and hepatitis.

#### 2.2.2. HIV

Another common viral infection that may infect the peripheral nervous system or CNS is HIV. The latter mainly causes sensory polyneuropathies.^52^ The mechanisms of neuropathy include interactions between viral proteins and nerve fibres, while indirect mechanisms include virus-mediated activation of glia and macrophage infiltration into the dorsal root ganglia.^9^ Neuropathic pain was the first clearly characterized chronic pain syndrome directly linked to HIV^39^ or its treatment, particularly older antiretroviral agents.^52^ It is often described as burning and associated with mechanical allodynia at the lower limbs with a distal characteristics distribution.^12,52^ HIV painful polyneuropathy tends to be less common than it was in early times while other types of chronic pain including widespread pain are now increasingly prevalent in HIV patients, with up to 50% suffering from chronic pain in their lifetime.^39^ Its treatment is similar to that of other neuropathic pain conditions.

#### 2.2.3. Enteroviruses

Specific enteroviruses, particularly enterovirus D68, which most commonly results in respiratory disorders, have recently been considered as a major cause of acute flaccid myelitis. Epidemic peaks of acute flaccid myelitis have been observed particularly in the United States every 2 years since 2012 coincident with peaks in enterovirus transmission, and a causal relationship is highly suspected.^34^ In addition to motor deficit, common symptoms at clinical evaluation include limb pain in a third of cases (in a cohort of 238 consecutive patients), which most probably corresponds to neuropathic pain.^34^ The long-term outcome is considered as generally favorable, but motor sequelae are possible and chronic pain has been reported after 1 year in 2 children of 8 affected with acute transverse myelitis.^44^

#### 2.2.4. Polioviruses

Polioviruses responsible for poliomyelitis have been eradicated in most parts of the world because of extensive vaccinations but are still prevalent in African, South American, or Asian countries. The disease causes permanent paralysis in 1 of 200 infections. As many as 60% to 80% of persons also develop chronic symptoms after poliomyelitis. These include muscle weakness, myalgia, joint pain but also, although less commonly, neuropathic pain^30^ and are generally referred to as postpolio syndrome. Pain is more frequent in women and in younger patients than in older people.^65^ Postpolio syndrome may be due to production of proinflammatory cytokines within the CNS and is particularly difficult to treat.^65^ Specific populations of patients including those with pain might benefit from immunoglobulins.^23^

#### 2.2.5. Tropical viruses

Chikungunya virus (CHIKV) is a mosquito-borne alphavirus that is endemic to several countries in Africa, South, Southeast Asia, and the Caribbean. Patients typically present with fever and rash, and up to 60% of them suffer from chronic pain particularly osteoarticular pain.^14^ However, neurologic symptoms are possible particularly CNS disease. Thus, myelitis has been reported in 22% of patients in a large-scale prospective study in Brazil.^8^ Chikungunya neurological complications infections may be responsible for neuropathic pain.^6,14^ A systematic cross-sectional study conducted in La Réunion in 2010 identified neuropathic pain in 19% of 104 consecutive patients with CHIKV. Pain with neuropathic characteristics was located mostly in the upper or lower limbs and was associated with more aggressive clinical picture, more impact in quality of life and more challenging pharmacological treatment.^14^

Zika is another tropical virus which is highly endemic in Brazil and may be associated with a large spectrum of neurologic syndromes.^8^ Zika is more often associated with PNS disease than Chikungunya, particularly Guillain–Barré syndrome (61% of patients in a large-scale prospective study).^8^

Human T-lymphotropic virus 1 (HTLV-1) generally induces myelopathy/tropical spastic paraparesis (HAM/TSP). Chronic pain including neuropathic pain is common but often neglected^32^ and has been described in Brazilian cohorts in up to 53% of cases.^49^ Neuropathic pain seems more common in patients carrying human T-cell lymphotropic virus type 1.^55^

### 2.3. Other coronaviruses and neurological complications

Similar to the SARS-CoV-2, other less common coronaviruses, including severe acute respiratory syndrome coronavirus 1 (SARS-CoV-1) with 8000 confirmed cases worldwide in 2002 to 2003 and Middle East respiratory coronavirus syndrome (MERS CoV) with a total of 2500 confirmed cases in the world since 2012, have been associated with neurological complications in severe cases.^60^ These include cerebrovascular pathologies and ischemic strokes, encephalitis, while rare cases of neuropathies, myopathies, and Guillain–Barré syndrome have been reported in SARS-CoV-1.^60^ However, no case of chronic pain has been reported after these infections, probably because outbreaks were limited in terms of number of cases and time.

## 3. Potential risk of COVID-19 in neuropathic patients

Since the onset of the pandemic in France in late January 2020, we have routinely followed 50 patients with chronic neuropathic pain caused by peripheral or central lesions (eg, PHN, chronic painful radiculopathy, diabetic painful neuropathy, spinal cord injury pain, and poststroke pain) exposed to the SARS-CoV-2. Although most of these patients (except 1 who died of COVID-19 respiratory complications) did not present with severe infection and were not hospitalized, they all reported a deterioration of their condition in terms of neuropathic pain symptoms for at least several weeks. Obviously explanations for enhanced neuropathic pain are multiple including psychological issues.^11^ However, given the high tropism of COVID-19 on the nervous system, we hypothesize that the neurotoxic consequences of this virus will be enhanced in patients with pre-existing neurological injury. Of note, a case of a severe ophthalmic acute herpes zoster and PHN has recently been reported as a complication of COVID-19 infection in an otherwise healthy 49 year-old woman.^62^ Presumably the fact that this patient was also infected with COVID-19 increased the risk of persistent neuropathic pain in this patient.

## 4. Neuropathic pain as a complication of COVID-19

Neuropathic pain may indirectly result from COVID-19 after ICU or may be caused by the SARS-CoV-2 itself.

### 4.1. Chronic neuropathic pain after intensive care unit in patients with COVID-19

The prevalence of persistent pain after ICU has been estimated to range from 28% to 77%.^40^ Persistent pain after ICU in patients with COVID-19 includes muscle pain related to joint contractures/muscle atrophy, or pain due to critical illness myopathy or polyneuropathy.^25^ Specific procedures used to treat severe acute respiratory distress syndrome may also induce tissue/nerve injuries. In particular, peripheral nerve injury associated with prone positioning deployed for improving oxygenation for management of acute respiratory distress syndrome has been reported in 14.4% of survivors of COVID-19 discharged to rehabilitation.^41^ These patients also spend significant time in the supine position while receiving neuromuscular blocking agents, which may increase their susceptibility to nerve injuries. Other potential causes of neuropathic pain after ICU include complications from traumatic procedures such as placement of chest tubes or tracheotomy.

### 4.2. Chronic neuropathic pain due to infection with COVID-19

Another potential mechanism for neuropathic pain after COVID-19 is the direct or indirect effect of the virus on the nervous system. Human coronaviruses are known to infect the peripheral nervous system or CNS through multiple mechanisms including cytokine secretions, general circulation of the virus, or direct invasion of the olfactory epithelium.^67^ Neurological complications of COVID-19 have been largely described in cohort studies or systematic reviews since their early descriptions in China.^18,24,26,42,48,66,67^ At the acute phase, they commonly include headache, dizziness, muscle pain, ataxia, and olfactory/taste disorders (anosmia and ageusia).^18,24,26,42,48,66,67^ These acute neurological complications have also be reported after other viral infections. However, although a number of viruses including influenza also gain entry through the olfactory bulb, olfactory or gustatory dysfunction is particularly common in patients infected with SARS-CoV-2.^1^ More severe complications have also been observed particularly in hospitalized patients; they include direct effects on the nervous system such as stroke, meningitis/encephalitis, and autoimmune disorders particularly Guillain–Barré syndrome and acute disseminated encephalomyelitis. Importantly, many of these neurological complications are at risk of neuropathic pain, in particular stroke, myelitis and Guillain–Barré syndrome. Possible neuropathic pain has been reported in up to 2.3% of hospitalized patients with COVID-19 in early series,^42^ but its prevalence is probably underestimated because it is well established that chronic neuropathic pain may also develop within months after injury to the nervous system.^12^

#### 4.2.1. Poststroke pain

Acute ischemic stroke has been reported in patients infected with SARS-CoV-2, although the risk seems low in hospitalized patients (0.9% according to a large recent meta-analysis).^58^ It may result from coagulation syndrome, myocarditis, or viral-induced vasculitis. Stroke may induce long-term neuropathic pain in 7% to 8% of patients within 1 year.^36^ Neuropathic poststroke pain may result from central disinhibition, sensitization, or thalamic changes and is particularly difficult to treat.^36^

#### 4.2.2. Neuropathic pain due to myelitis

Acute transverse myelitis has been reported in several cases of patients with COVID-19 and may result from immune complication or directly relate to viral invasion.^3,10,56,63^ Myelitis may be responsible for central neuropathic pain at level or below level, as is the case for most spinal cord injury lesions. One case report described a COVID-19 female patient with intense chronic burning pain involving an area innervated by multiple levels of spinal nerves, potentially resulting from myelitis.^2^

#### 4.2.3. Neuropathic pain associated with Guillain–Barré syndrome

To date, multiple case reports of Guillain–Barré syndrome have been published in patients with COVID-19 particularly in the United Kingdom, Italy, or China.^50^ In most cases, the symptoms were noted to occur within days of the COVID-19 infection. However, unlike typical Guillain–Barré syndrome (GBS), most patients were elderly and had concomitant respiratory complications. In several case reports, GBS has been noted to occur 2 to 3 weeks after the onset of infection and after recovery^4,57^ and was not necessarily preceded by respiratory symptoms or fever. This pattern corresponds to the classic postinfectious pattern, also observed for other viral infections such as Zika or other coronaviruses, and suggests autoimmune response. Thus far, the most common pain-related symptom after COVID-19–induced GBS has been myalgia.^50^ However, GBS often causes acute neuropathic pain, mainly through impairment of small nociceptive fibers,^45^ and chronic neuropathic pain has also been reported in severe cases.

## 5. Implications for therapeutic management

Neuropathic pain should be distinguished from other causes of COVID-19–induced pain because it is more difficult to treat.^19,47^ Although conventional analgesics are not effective and not recommended, a number of patients with neuropathic pain, particularly elderly patients, receive or self-administer these medications for their pain particularly nonsteroidal anti-inflammatory agents.^46^ Multiple concerns have been raised about the use of nonsteroidal anti-inflammatory agents in patients infected with SARS Co-2, but recent large-scale surveys seem to indicate that their use is not associated with significant increase in mortality, hospitalization, or ICU admission.^38^ The mainstay of therapy for neuropathic pain is represented by gabapentinoids (gabapentin and pregabalin), antidepressants (serotonin and noradrenalin reuptake inhibitor antidepressants or tricyclic antidepressants), tramadol, and topical agents (lidocaine plasters, capsaicin high concentration patches or botulinum toxin A for peripheral neuropathic pain), while strong opioids may be considered in refractory cases.^19,47^ However, these drugs have an overall modest therapeutic efficacy.^19,47^ Nonpharmacological treatments including invasive or noninvasive neurostimulation techniques (transcutaneous electrical nerve stimulation, repetitive transcranial magnetic stimulation, spinal cord stimulation, etc) may also be proposed, although robust evidence for their efficacy still needs adequate large-scale controlled trials.^47^ The reported female patient affected with COVID-19 who reported intense burning pain responded to gabapentin.^2^

## 6. Summary/future directions

Considering the importance of neurological complications of COVID-19, we anticipate that a number of patients infected with COVID-19 will develop neuropathic pain within weeks or months or that patients with neuropathic pain will present with deterioration of their neurological complication or exacerbation of their pain. As for now, we do not have consistent data regarding the prevalence and clinical characteristics of neuropathic pain in patients infected with COVID-19. However, several prospective studies are underway in France in particular in our hospital group in patients previously admitted in ICUs (Martinez et al. in preparation). It is also probable that chronic neuropathic pain affects less severe COVID-19 patients. We need prospective cohort studies of these patients because neuropathic pain may considerably affect quality of life and should therefore be detected as early as possible for adequate management. Interestingly, prospective web-based surveys have recently been conducted in Canada to identify the risks of the COVID-19 pandemic in patients with chronic pain (this special issue). Web-based cohorts of painful patients followed in pain clinics which are currently being implemented in France (www.institut-analgesia.org) should also be helpful in this respect. Finally, it will be of interest to prospectively follow patients with chronic pain affected with COVID-19 and determine whether the risk of pain exacerbation is distinct in neuropathic compared with non-neuropathic patients.

## Disclosures

The authors report no conflicts of interest related to the publication.
